# A Self-Sensing Piezoelectric MicroCantilever Biosensor for Detection of Ultrasmall Adsorbed Masses: Theory and Experiments

**DOI:** 10.3390/s130506089

**Published:** 2013-05-10

**Authors:** Samira Faegh, Nader Jalili, Srinivas Sridhar

**Affiliations:** 1 Piezoactive Systems Laboratory, Department of Mechanical and Industrial Engineering, Northeastern University, 360 Huntington Ave., Boston, MA 02115, USA; E-Mail: faegh.s@husky.neu.edu; 2 Nanomedicine Science and Technology Center, Department of Physics, Northeastern University, 360 Huntington Ave., Boston, MA 02115, USA; E-Mail: s.sridhar@neu.edu

**Keywords:** microcantilever, biosensor, distributed-parameter modeling, mass detection

## Abstract

Detection of ultrasmall masses such as proteins and pathogens has been made possible as a result of advancements in nanotechnology. Development of label-free and highly sensitive biosensors has enabled the transduction of molecular recognition into detectable physical quantities. Microcantilever (MC)-based systems have played a widespread role in developing such biosensors. One of the most important drawbacks of all of the available biosensors is that they all come at a very high cost. Moreover, there are certain limitations in the measurement equipments attached to the biosensors which are mostly optical measurement systems. A unique self-sensing detection technique is proposed in this paper in order to address most of the limitations of the current measurement systems. A self-sensing bridge is used to excite piezoelectric MC-based sensor functioning in dynamic mode, which simultaneously measures the system's response through the self-induced voltage generated in the piezoelectric material. As a result, the need for bulky, expensive read-out equipment is eliminated. A comprehensive mathematical model is presented for the proposed self-sensing detection platform using distributed-parameters system modeling. An adaptation strategy is then implemented in the second part in order to compensate for the time-variation of piezoelectric properties which dynamically improves the behavior of the system. Finally, results are reported from an extensive experimental investigation carried out to prove the capability of the proposed platform. Experimental results verified the proposed mathematical modeling presented in the first part of the study with accuracy of 97.48%. Implementing the adaptation strategy increased the accuracy to 99.82%. These results proved the measurement capability of the proposed self-sensing strategy. It enables development of a cost-effective, sensitive and miniaturized mass sensing platform.

## 1. Introduction

Microcantilever (MC) sensors have emerged as a new technology for detecting ultrasmall masses and biological species. Detection of proteins and pathogens, physical parameters [1,2] and biochemical agents [3–10] has been demonstrated utilizing these sensors. MC sensors' sensitivity and capability in detecting small forces, mechanical stresses, and added adsorbed mass molecules, have generated widespread interest in using this type of sensing in different applications [11–15]. One of the most inspiring applications of MC sensors has been their implementation as an inexpensive, sensitive, label-free platform for real-time detection of biomolecules [16–24]. Multiplexed detection of concentrations of antigens in a sample fluid has also been enabled utilizing arrays of MCs.

MC-based biosensors operate in two main modes: (*i*) static mode and (*ii*) dynamic mode. Most of the studies regarding identification of molecular affinities have been performed in the static mode where the induced surface stress as a result of deflection of MC from a stable baseline was used to measure molecular binding [17,18,23,25–35]. Arrays of MCs have been used for high-throughput measurements [16,21,24,25,33–35]. On the other hand, in dynamic mode, the system is brought into excitation at or near its resonance frequency. The shift in the resonance frequency as a result of molecular recognition yields a good insight into the amount of adsorbed mass [36,37].

There are two main features determining the success of all biological sensors: first, the molecular binding between the receptor and the biomolecule of interest; second, the read-out system capable of transducing the molecular binding into detectable physical property. There are a number of read-out methodologies including optical-based, capacitive-based, piezoresistive-based and piezoelectric-based measurement techniques. Optical measurement systems are the most commonly-used and powerful technique in measuring variations at the micro- and nano-scale. They are widely used in atomic force microscopy (AFM). The main challenges of this technology are high cost, surface preparation, and optical alignment and adjustment requirement. Moreover, miniaturizing the detection platform and implementing in non-transparent solutions and liquid chambers are not possible with this methodology.

Capacitive-based MC sensors monitor capacitance change as a result of deflection of MC. There are some limitations with this type of sensing technology which include low resolution, complicated electronic circuits and fabrication processes. Piezoresistive MC sensors work based on change of resistance in the piezoresistive layer when MC deforms. The change of resistance can be measured utilizing the output voltage of the system. However, there is difficulty in fabricating the sensor with the embedded resistor. Another measurement method is piezoelectric-based systems where a piezoelectric material is used in order to create voltage as a result of induced surface stress due to mechanical deformation of the beam. This technique provides a simple sensitive read-out mechanism.

**Figure 1.**
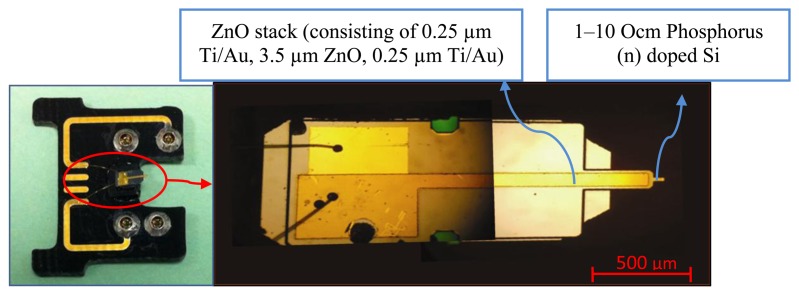
Veeco Active Probe with ZnO self-sensing layer deposited on the probe.

In order to overcome the aforementioned challenges, a unique self-sensing piezoelectric-based MC sensor is proposed and studied in this paper. In self-sensing MC sensors both direct and inverse properties of a piezoelectric material is utilized to play the role of both sensor and actuator. Direct piezoelectric property is used to sense the self-induced voltage generated in the piezoelectric layer as a result of beam deformation. At the same time, inverse property of piezoelectric material is used to generate deformation and bring the system into vibration as a result of applying a harmonic voltage to it. Therefore, a single piezoelectric layer embedded in the MC sensor is utilized to both actuate and sense the system exploiting a capacitance bridge network. This provides a simple and inexpensive platform for mass sensing and detection purposes with the opportunity of miniaturizing the platform. A Veeco Active Probe [38] is used here where a ZnO stack is used to implement the MC in self-sensing mode as shown in Figure 1.

Although MC-based biosensors have received a widespread attention for label-free bio-detection, there are not enough analytical studies investigating modeling and simulation of piezoactive MC-based biosensors. Most of the related studies are based on simple lumped-parameters system modeling the biosensor using Euler-Bernoulli beam theory [31,39,40]. Finite Element Method (FEM) has been extensively implemented for numerically modeling MC based systems [41–50]. It has emerged as a promising tool for estimating geometry and bending stiffness of MCs [46], identifying material and geometrical parameters of microstructures [47], verification of analytical models [48] and fabrication [49] of MCs. 3D dynamic behavior of an eight cantilever array structure was analyzed numerically by AFM showing good agreement in lower mode but not in higher modes [50]. However such systems (lumped-parameters modeling) and such numerical analyses are not capable of describing all dynamics and phenomena occurring within the cantilever with any type of designs and geometries and in all vibrational modes. Therefore, there is still a need for a more comprehensive mathematical framework capable of describing static and dynamic behavior of MCs. Therefore, in the first part of this paper mathematical modeling is developed for self-sensing piezoelectric-based MC followed by simulation results.

In the final part of the paper, an experimental setup is developed and extensive testing is performed on Veeco Active Probe equipped with piezoelectric layer functioning in dynamic mode. A capacitance bridge network is utilized to implement the active probe in self-sensing mode. Detection of adsorbed biological species, which is the covalent binding of thiol gourps of Aminoethanethiol, was made possible through the proposed self-sensing piezoelectric-based MC sensor. Similar mass detection setup was built and performed utilizing optical-based method and the results were compared to the self-sensing methodology to verify the applicability of the proposed platform. Quantification of adsorbed masses was carried out and the sensitivity of the system was measured.

Piezoelectric properties at the nanoscale are sensitive to temperature and other ambient variations. In order to have a precise model of the actuation/sensing, an adaptation strategy needs to be implemented in order to compensate for the variation of piezoelectric property (here ZnO stack). For this a mathematical adaptation law is presented in Section 3 followed by simulation results and comparison with those of Section 2. The experimental results were verified with the theories presented in Sections 2 and 3. Based on the results, the accuracy of the proposed modeling frameworks is demonstrated.

## 2. Mathematical Modeling

### 2.1. Beam Modeling

**Figure 2.**
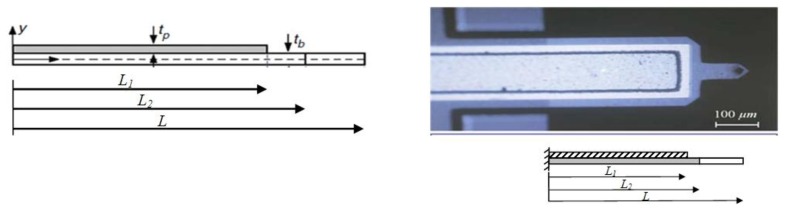
Micrograph/photograph of a Veeco Active Probe with a ZnO stack on top extended from 0 to *l_1_* [53].

A comprehensive mathematical modeling is proposed in this section using a distributed-parameters model. The system includes a Veeco Active Probe as a self-sensing MC in dynamic mode. The MC beam is assumed to obey the Euler-Bernoulli beam theory. The use of Euler-Bernoulli beam theory was proven to model the current system with a high level of accuracy compared to plate theory in earlier work of the authors [51]. The self-sensing mode can be implemented through the ZnO stack mounted on the base of the probe extending close to the tip as shown in Figure 2. The MC beam is narrowed in the tip which adds to the sensitivity of the system. The MC beam is modeled as a nonuniform cross-section beam with the total length of *L* and an active length (piezoelectric layer) of *L_1_* which is used for functionalization. The beam is considered to have thickness *t_b_*, and volumetric density *ρ_b_*. The piezoelectric layer over the top surface of the cantilever has thickness *t_p_*, and volumetric density *ρ_p_*. Both MC beam and piezoresistive layer are considered to have width *b* [52].

*w*(*x,t*) denotes the midplane deflection of MC beam with the tip deflection as *w*(*L,t*). Small deflection and linear system properties assumptions are taken into account. The equation of motion of the system is derived using Extended Hamilton's principle. The system is typically actuated by applying a harmonic sinusoidal voltage to the piezoelectric layer.

The following distributed-parameters modeling is proposed for the response of the system to the applied input. For this, the kinetic energy of the system is written as:

(1) $$KE=\frac{1}{2}\int_{0}^{L}\rho \;(x){\left[\frac{\partial w\;\left(x,t\right)}{\partial t}\right]}^{2}dx$$

where:

(2) $$\rho \;\left(x\right)=\rho A\;(x)=\rho_{b}bt_{b}+\rho_{p}bt_{p}\;G\left(\text{x}\right)$$

with *G*(*x*) = 1 − *H*(*x* − *L_1_*), and *H*(*x*) being the Heaviside function. Considering that beam only extends in the *x*-direction, potential energy of the system is written as:

(3) $$\delta PE=\int_{0}^{L}\sigma_{x}\;\delta \in_{x}dx$$

in which the stress-strain relationship for beam and piezoelectric layer can be obtained from:

(4) $$\sigma_{x}^{b}=E_{b}\;{ɛ}_{\text{x}}$$

(5) $$\sigma_{x}^{p}=E_{p}\;{ɛ}_{\text{x}}+E_{p}\;{\text{d}}_{31}\frac{\text{V}(\text{t})}{{\text{t}}_{\text{p}}}$$

with *E_b_* and *E_p_* being beam and piezoelectric elastic moduli, respectively. *V*(*t*) is the applied input voltage to the system, and *d_31_* is the piezoelectric constant [54]. Strain in the *x*-direction is related to the transverse deflection of the beam by ${ɛ}_{\text{x}}=-y\frac{\partial^{2}\text{w}\;(\text{x},\text{t})}{\partial {\text{x}}^{2}}$ which should be modified as ${ɛ}_{\text{x}}=-(y-{\text{y}}_{\text{n}})\frac{\partial^{2}\text{w}\;(\text{x},\text{t})}{\partial {\text{x}}^{2}}$ when used for piezoelectric section as a result of shift in the neutral axis. This shift *y_n_* can be expressed as:

(6) $$y_{n}=\frac{E_{p}\;t_{p}\;(t_{p}+t_{b})}{2\;(E_{p}\;t_{p}+E_{b}\;t_{b})}$$

Therefore, the virtual potential energy can be rewritten as:

(7) $$\delta PE=\int_{0}^{L}\frac{\partial^{2}}{\partial {\text{x}}^{2}}\;[EI\;\left(x\right)\frac{\partial^{2}\text{w}\;(\text{x},\text{t})}{\partial {\text{x}}^{2}}]dx+M_{p0}V\;(t)\int_{0}^{L_{1}}\frac{\partial^{2}\text{G}\;(\text{x})}{\partial {\text{x}}^{2}}dx$$

where *M_p0_* is defined as:

(8) $$M_{p0}=bE_{p}\;d_{31}\left[\frac{1}{2}(t_{b}+t_{p})-y_{n}\right]$$

The varying stiffness of the system *EI*(*x*) is:

(9) $$\begin{array}{c} EI\;\left(x\right)=E_{b}\;I_{b}\;\left(x\right)+E_{p}\;I_{p}\;\left(x\right)\\ I_{b}\;\left(x\right)=\frac{1}{12}bt_{b}^{3}+G\;\left(x\right)bt_{b}\;y_{n}^{2}\\ I_{p}\;\left(x\right)=\left[\frac{1}{12}bt_{p}^{3}+bt_{p}\;y_{n}^{2}\;{\left(\frac{1}{2}\;(t_{b}+t_{p})-y_{n}\right)}^{2}\right]bG\;(x) \end{array}$$

The virtual work due to ever-present viscous and structural damping terms is given by:

(10) $$\delta W=\int_{0}^{L}\;(-B\dot{w}\;(x,t)-C{\dot{w}}^{\prime }\;(x,t))\;\delta w\;(x,t)\;dx$$

where *B* and *C* represent the coefficients of viscous and structural damping, respectively [55]. ()′ is partial derivative with respect to spatial coordinate *x*, while ()˙ denotes temporal derivative.

**Figure 3.**
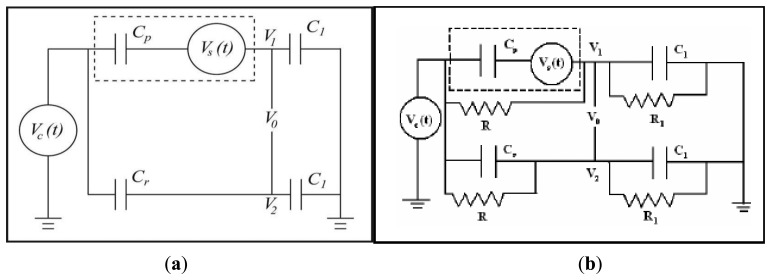
(**a**) Pure capacitive bridge, and (**b**) Resistive-Capacitive (R-C) bridge [53].

Two main impedance bridges have been used to supply voltage and sense the induced voltage in the piezoelectric patch [56]. They are mainly pure capacitive and resistive-capacitive bridges as shown in Figure 3.

The piezoelectric actuator is modeled as a capacitor and a voltage source in series as shown in the dashed box in Figure 3. *C_p_* represents the effective capacitance of the piezoelectric element and *V_s_* is the induced voltage in the piezoelectric patch. For the purpose of self-sensing, the piezoelectric actuator is connected in a bridge with other elements (*i.e.*, the capacitors *C_1_*, *C_r_* and/or resistors *R*, *R_1_*). In this study, pure capacitive bridge network in employed as shown in Figure 3(a). *V_c_*(*t*) is the voltage applied across the capacitor bridge. Therefore, the voltage applied across the piezoelectric actuator can be written as:

(11) $$V\left(t\right)=\frac{C_{1}}{C_{1}+C_{p}}V_{c}\;\left(t\right)-\frac{C_{p}}{C_{1}+C_{p}}V_{s}\;(t)$$

The self-induced voltage generated in the piezoelectric layer as a result of induced surface stress due to beam vibration can be written as [53]:

(12) $$V_{s}\;(t)=\frac{1}{C_{p}}bE_{p}\;d_{31}\;(\frac{1}{2}(t_{b}+t_{p})-y_{n})\;\left[w^{\prime }\;(L_{1},t)-w^{\prime }\;(0,t)\right]$$

Substituting Equation (12) into (11) and then into (7) and implementing Extended Hamilton's principle, the equation of motion of the system can be derived as:

(13) $$\begin{array}{c} \rho \;(x)\;\frac{\partial^{2}w\;(x,t)}{\partial t^{2}}+\frac{\partial^{2}}{\partial x^{2}}\left[EI\left(x\right)\;\frac{\partial^{2}w\;(x,t)}{\partial x^{2}}\right]+B\;\frac{\partial w\;(x,t)}{\partial t}+C\frac{\partial^{2}w\;(x,t)}{\partial x\partial t}-\frac{M_{p0}}{C_{p}\;+\;C_{1}}\;bE_{p}\;d_{31}\\ \left(\frac{1}{2}(t_{b}+t_{p})-y_{n}\right)\;\left[\;w^{\prime }\;(L_{1},t)-w^{\prime }\;(0,t)\right]\;G^{\prime }\;(x)=-M_{p0}\;V_{c}\left(t\right)\;G^{\prime }\;(x) \end{array}$$

with the boundary conditions:

(14a) $$w\;(0,t)=w^{\prime }\;(0,t)=0$$

(15) $$w^{\prime }\left(L,t\right)=w^{\prime \prime \prime }\left(L,t\right)=0$$

The self-sensing nature appears in the equation of motion such that *V_c_*(*t*) appearing in the right hand side of the equation is employed for actuation, and at the same time, the sensing effect is observed in the left hand side (with the extra term being a function of slope of the beginning and end points of piezoelectric layer).

That is, from Equation (12), it is observed that the voltage generated in the piezoelectric layer, *V_s_*(*t*), is a function of the slope of the beginning and end point location of the piezoelectric layer which contains the information of the response of the system. In order to acquire this signal, its introduction into the output voltage of the capacitive bridge should be analyzed. For this, the bridge output voltage is expressed as [56]:

(16) $$V_{0}\;\left(t\right)=\left[\frac{C_{p}}{C_{1}+C_{p}}-\frac{C_{r}}{C_{1}+C_{r}}\right]V_{c}\;\left(t\right)+\frac{C_{p}}{C_{1}+C_{p}}V_{s}\;(t)$$

In order to extract the induced voltage from the bridge output signal, the bridge should be balanced by selecting the appropriate bridge elements such as *C_1_* and *C_r_*. Frequency analysis of the obtained self-induced signal would reveal information about the resonance frequencies of the system. Being able to have an insight into the resonance frequencies of the system, the effect of adsorbed mass on the MC surface can be analyzed running the system in dynamic mode.

### 2.2. Numerical Simulation and Results

In order to solve the obtained governing equation of motion [Equation (13)], it is discretized using Galerkin's method [56]. For this, the partial differential Equation (13) is converted into ordinary differential equation (ODE) using the following discretization:

(17) $$w\;\left(x,t\right)=\sum_{j=1}^{n}\;\phi_{j}\;(x)q_{j}\;(t),\;j=1,2,\ldots ..,n$$

with *φ_j_*(*x*) and *q_j_*(*t*) being the clamped-free beam eigenfunction and generalized coordinates, respectively. Therefore, the equation of motion can be expressed as a function of time in a matrix form. The ODE for the system can be then represented as:

(18) $$M\ddot{q}\;(t)+D\dot{q}\;(t)+Kq\;(t)=K_{v}V\;(t)$$

where

(19) $$\begin{array}{c} q=\left\{q_{1},q_{2},\ldots .q_{i}\right\},\dot{q}=\left\{{\dot{q}}_{1},{\dot{q}}_{2},\ldots .{\dot{q}}_{i}\right\}\\ M=\left\{M_{ij}\right\},\;M_{ij}=\int_{0}^{L}\rho A\;(x)\;\phi_{j}\;\left(x\right)\;\phi_{i}\left(x\right)\;dx,i,j=1,2,\ldots ..,n\\ D=\left\{D_{ij}\right\},D_{ij}=B\int_{0}^{L}\;\phi_{j}\;\left(x\right)\;\phi_{i}(x)\;dx+C\int_{0}^{L}\phi_{j}^{\prime }\;(x)\;\phi_{i}\;\left(x\right)dx\\ K=\left\{K_{ij}\right\},\;K_{ij}=\int_{0}^{L}EI\;(x)\;\phi_{j}^{\prime \prime }\;\left(x\right)\;\phi_{i}^{\prime \prime }\;(x)\;dx\\ -\frac{M_{P0}}{C_{1}\;+\;C_{p}}K_{s}\;\left[\phi_{j}^{\prime }\;\left(L_{1}\right)-\phi_{j}^{\prime }\;\left(0\right)\right]\;\left[\phi_{i}^{\prime }\;\left(L_{1}\right)-\phi_{i}^{\prime }\;\left(0\right)\right]\\ K_{v}=\left\{K_{vj}\right\},K_{vj}=-M_{p0}\int_{0}^{L}\phi_{j}^{\prime }\;\left(x\right)\;\delta (x-L_{1})\;dx=-M_{p0}\phi_{j}^{\prime }\;(L_{1}) \end{array}$$

System parameters used for simulation are listed in Table 1. The forced vibration problem represented by ODE (17) was solved in Matlab with the input being the applied voltage to the ZnO stack mounted on active probes.

A harmonic voltage with the amplitude of 2.5 V and frequency close to system's first natural frequency was applied and the system's generalized coordinates for at least two modes, *q*_1_(*t*) and *q*_2_(*t*) were obtained. *φ_i_*(*x*) was selected to be the admissible function of a clamped-free beam with the modified mass and stiffness properties of a beam with piezoelectric layer. The values of *C_1_* and *C_r_* were selected to be 30 pF. The deflection of MC with respect to location and time, *w*(*x*,*t*) was calculated using Equation (16).

**Figure 4.**
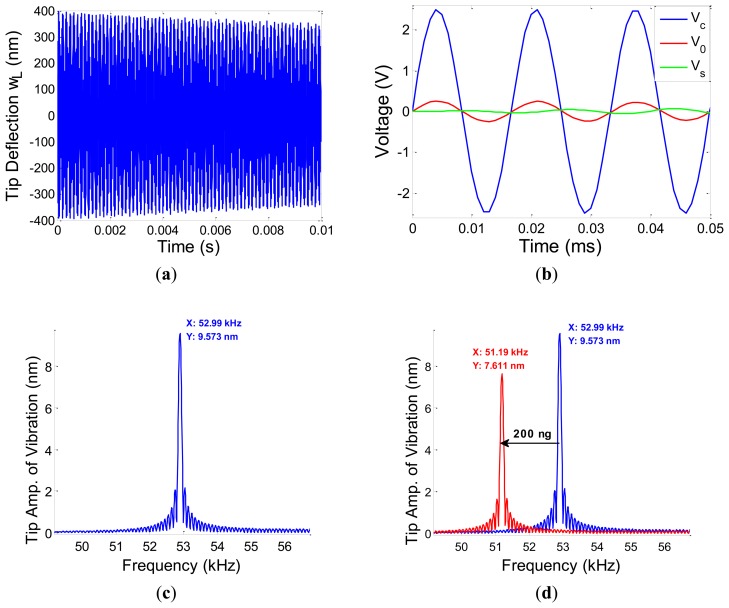
Numerical results: (**a**) tip deflection of microcantilever, *w* (*L*,*t*), (**b**) Input voltage, *V_c_*(*t*), output voltage, *V_0_*(*t*), and self-induced voltage, *V_s_*(*t*), (**c**) FFT response of the system with 1st natural frequency highlighted, (**d**) the effect of added surface mass due to functionalization on the first natural frequency.

Consequently, deflection of the tip of the cantilever, *w*(*L*,*t*), output voltage, V_0_(*t*), and self-induced voltage, *V_S_*(*t*) were obtained and plotted in Figure 4(a,b). Taking Fast Fourier Transform of the system's response, the first natural frequency of the system was obtained to be 52.99 kHz as illustrated in Figure 4(c). The effect of ultra-small adsorbed mass was modeled as added surface mass over the gold-coated MC surface, 0-*L_1_*. The amount of adsorbed mass was assumed to be as low as 200 ng which resulted in the reduction of the 1st natural frequency to the amount of 1.8 kHz. The shift in natural frequency is depicted in Figure 4(d).

**Figure 5.**
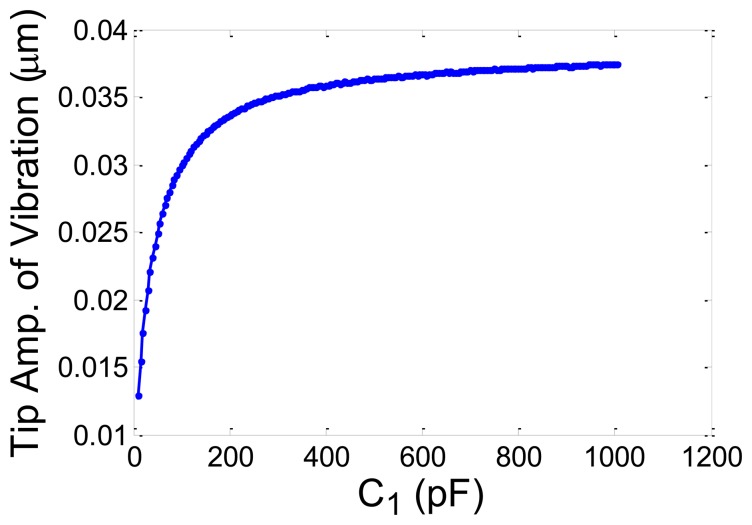
Sensitivity of the vibration amplitude of the tip of MC with respect to *C_1_*.

Sensitivity of vibration amplitude of the MC tip with respect to the selected *C_1_* was studied and it was shown that the amplitude of tip vibration increased by increasing the value of *C_1_* as shown in Figure 5.

## 3. Adaptive Estimation

### 3.1. Adaptation Law

Considering the fact that the properties of the piezoelectric material vary with ambient temperature and time, compensating for these variations would dynamically improve the proposed self-sensing implementation. An adaptive compensatory self-sensing strategy [57] is utilized in order to estimate the variations of the capacitance of piezoelectric material, *C_p_*, with respect to time.

In order to compensate for time variation of *C_p_*, a parameter *θ* is defined which is the ratio of impedances in the bridge as follows:

(19) $$\theta =\frac{C_{p}}{C_{1}+C_{p}}$$

**Figure 6.**
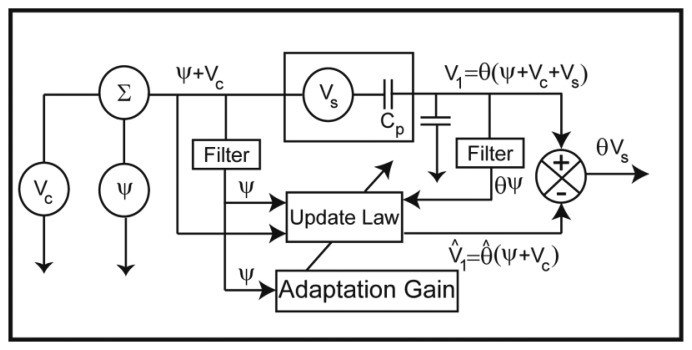
Schematic of the adaptive self-sensing strategy.

The estimation of the measured parameter *θ* is defined to be *θ̂* which needs to be obtained. In order to find the estimated parameter, *θ̂*, a parametric error is defined as *θ̃* = *θ* − *θ̂* which should be driven to zero. Figure 6 shows the schematic of the adaptive self-sensing strategy. *Ψ*(*t*) is a low power persistent excitation signal which is applied to measure. *θ* It should be low enough such that it does not introduce vibration in the MC and contribute to the self-induced voltage.

Referring to Figure 6, the voltage of the upper branch of the bridge can be written as:

(20) $$V_{1}=\theta \psi \;(t)$$

with *V̂;*_1_ being the estimation of *V*_1_ as:

(21) $$\hat{V_{1}}=\hat{\theta }\psi \;(t)$$

Therefore, the bridge output voltage can be expressed as:

(22) $$V_{0}\;\left(t\right)=V_{1}\;\left(t\right)-\hat{V_{1}}\left(t\right)=\left(\theta -\hat{\theta }\;\right)\psi \;(t)=\tilde{\theta }\psi \;(t)=e\;(t)$$

The proposed adaptation law for estimation of parameter *θ* is as follows [57]:

(23) $$\dot{\hat{\theta }}\;\left(t\right)=-k_{1}e_{1}-P_{1}\;\left(t\right)\;\frac{\partial }{\partial \hat{\theta }}\;e_{1}^{2}$$

which can be further simplified as:

(24) $$\dot{\hat{\theta }}\;\left(t\right)=-k_{1}\;\psi \tilde{\theta }-P\;(t)\;\psi^{2}\tilde{\theta }$$

where *k_1_* and *P*(*t*) represent a constant gain and time dependent adaptation gain respectively. The time-varying adaptation gain can be replaced by a constant gain in order to simplify the calculation. Therefore, the update law can be simplified to:

(25) $$\dot{\hat{\theta }}\;\left(t\right)=-k_{1}e_{1}-P_{0}\frac{\partial }{\partial \hat{\theta }}e_{1}^{2}$$

and consequently:

(26) $$\dot{\hat{\theta }}\;\left(t\right)=-k_{1}\psi \;\tilde{\theta }-P_{0}\psi^{2}\;\tilde{\theta }$$

where *P_0_* represents the constant adaptation gain (*P_0_* > 0). References [56] and [57] provide more information regarding the implementation of adaptation law.

### 3.2. Simulation Results for Adaptive Estimation

**Figure 7.**
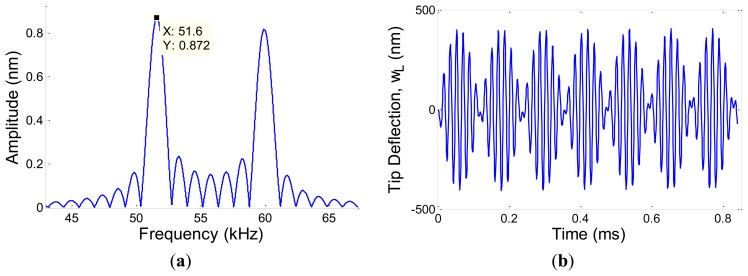
(**a**) Tip deflection of MC, *w_L_*(*x*,*t*), (**b**) FFT response of the system with 1st natural frequency highlighted.

In this section, the equation of motion presented in Section 2.1. is simulated considering the estimated time varying piezoelectric capacitance, *C_p_* obtained through implementing adaptive estimation strategy. All other conditions are kept the same as those in Section 2.1. System's response along the beam at any time, *w*(*x*,*t*) is obtained. Consequently tip deflection and frequency response of the system are calculated and plotted as depicted in Figure 7.

**Figure 8.**
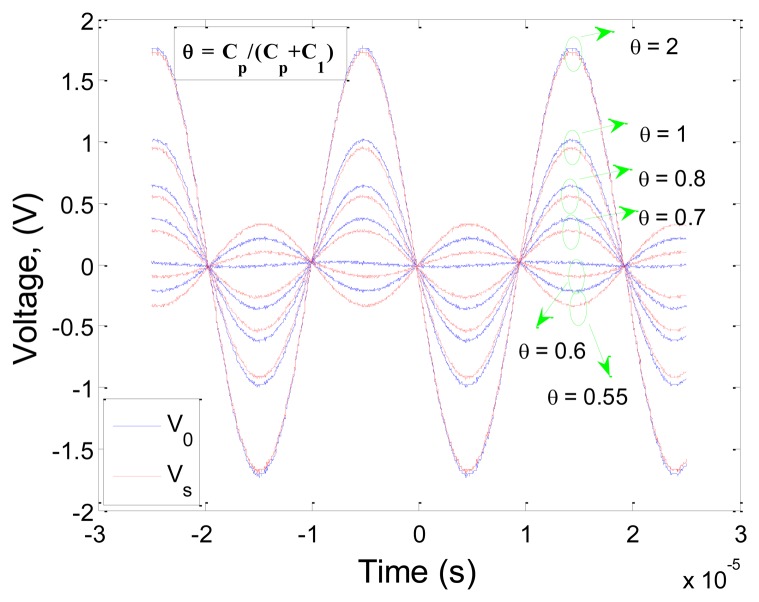
The effect of *θ* on the calculation of self-induced voltage, *V_s_*(*t*).

It is shown that the first natural frequency of the system is obtained to be 51.6 kHz, which is about 1.3 kHz less than that obtained in Section 2.2. The contribution of the self-induced voltage in the bridge output signal is dependent on the unknown gain defined as *θ*. A study was conducted to investigate the effect of the defined *θ* on the reasonable and maximum contribution of the *V_s_*(*t*). The result is depicted in Figure 8 showing that by increasing *θ*, the calculated self-induced voltage gets closer to the output voltage.

## 4. Experimental Setup

In this section, the capability of the self-sensing strategy is tested experimentally and the results are compared with those obtained from the mathematical modeling presented in Sections 2 and 3. The same experiment was performed using a laser vibrometer as the read-out method to verify the self-sensing measurement technique.

**Figure 9.**
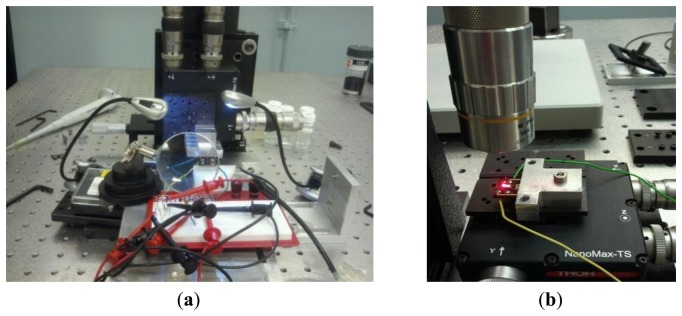
Veeco Active Probe mounted on a holder (**a**) connected to the pure capacitive bridge for self-sensing implementation, (**b**) placed under laser vibrometer head.

A Veeco Active Probe [38] was implemented with the self-sensing capability. A pure capacitive bridge (Figure 3(a)) was used to send a harmonic voltage to the ZnO stack mounted at the base of each probe and at the same time receive the output voltage as a result of MC vibration. The Active Probe was mounted on a holder which was fixed on a 3D stage. Figure 9(a) shows the experimental setup for implementing self-sensing strategy. The value of *θ* = 0.5 was used experimentally. The same platform is placed under the laser vibrometer (Polytec CLV-2534) in order to measure MC vibrations through optical method as shown in Figure 9(b).

### 4.1. Non-Functionalized MC: Verification with Modeling

**Figure 10.**
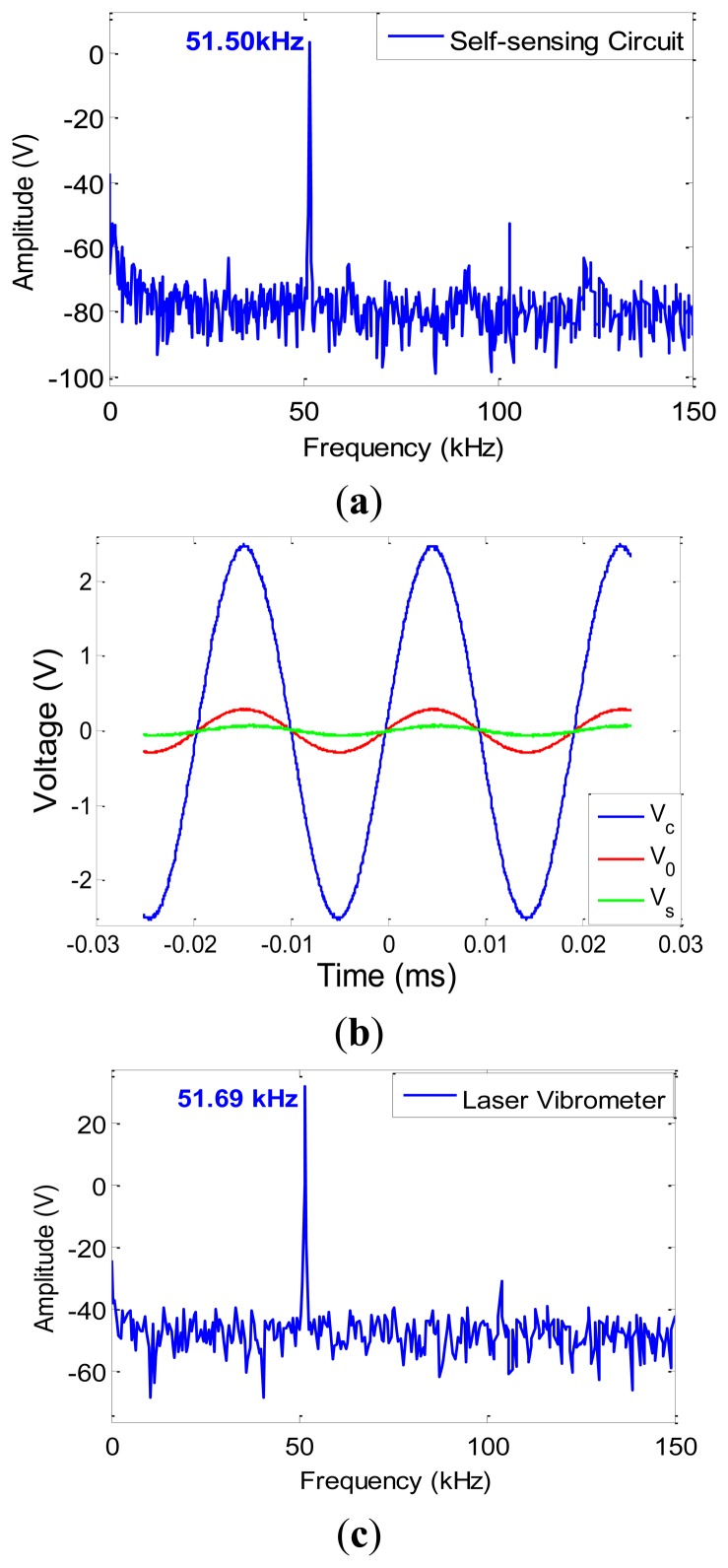
(**a**) FFT of the response of the system using self-sensing bridge, (**b**) Input, output and self-induced voltages, (**c**) FFT of the response of the system using laser vibrometer.

Measurement of the first resonance frequency of a non-functionalized MC was made in this section implementing both self-sensing strategy and optical read-out systems. In order to obtain the frequency at which the system resonates, the excitation frequency of system's input was swept from 0 kHz to 100 kHz with resolution of 10 Hz. The amplitude was kept at 2.5 V. Taking FFT of the output voltage obtained from the bridge, it was observed that the first resonance frequency of the non-functionalized MC was captured at 51.50 kHz which is in a great level of accuracy with the theoretical result obtained through implementing adaptive strategy. Having the input voltage, *V_c_*(*t*), and measuring the output voltage, *V_0_*(*t*), through the self-sensing bridge and consequently calculating self-induced voltage, *V_s_*(*t*), relatively similar results were obtained compared to the theoretical part. Figure 10(a) shows the FFT of the response of the system while Figure 10(b) shows input, output and self-induced voltages.

Performing the same experiment through a laser vibrometer, the resonance frequency of the system was captured to be 51.69 kHz which proves the capability of the self-sensing strategy with the precision of 99.63%. The obtained frequency response is depicted in Figure 10(c). Table 2 shows a comparison between the experimental results to theoretical ones obtained from Sections 2 and 3.

### 4.2. Functionalized MC: Detection of Adsorbed Mass

In this section the main application of the developed platform is tested. A Veeco Active Probe equipped with a self-sensing read-out mechanism was implemented to detect the adsorbed mass over the gold surface. The system was operated in dynamic mode where the MC was brought into excitation by applying a harmonic voltage to the self-sensing bridge with a frequency close to system's first resonance frequency.

Thiol groups which attach to many biomolecules were immobilized over the MC surface by making a covalent binding to gold creating a self-assembled monolayer. The gold-coated surface was washed in acetone, ethanol and DI water for 10 minutes. The main challenge in functionalizing the self-sensing active probe is the integrated electronics on the base of the probe. Therefore, washing and submerging it into any solution comes with the risk of damaging or destroying the whole platform. In order to address this issue, a Teflon chamber was designed such that creating any droplet of liquids over the chamber's surface was made possible. The Active Probe was then mounted on a holder and placed over a 3D stage with resolution of submicron which was used to place MC tip into the droplet such that it does not wet any electronics in the vicinity of the probe.

A 0.1 M of aminoethanethiol solution was prepared by dissolving 2-aminoethanethanethiol powder in deionized water. The tip of the MC was dipped into a droplet of the prepared solution. As a result, self-assembled monolayer of aminoethanethiol was formed over the gold surface by attachment of thiol groups to gold.

**Figure 11.**
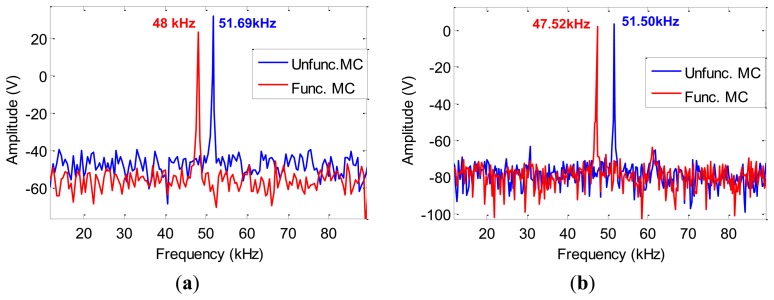
Shift in the first resonance frequency measured by (**a**) self-sensing bridge, (**b**) Laser vibrometer.

In order to find the frequency at which the system resonates after functionalization, the excitation frequency was swept between 0-100 kHz. The response of the system was measured by both self-sensing bridge and the laser vibrometer as shown in Figure 11. The amount of shift in the first resonance frequency of the system was observed to be equal to 3.98 kHz and 3.69 kHz as obtained from self-sensing bridge and laser vibrometer, respectively. Measurements were performed multiple times and a frequency sweep was carried out each time. The standard deviation was calculated to be 0.2098. The results obtained by the laser vibrometer reinforce those obtained by the self-sensing bridge. However, there are certain limitations with implementing laser vibrometer measurements in liquid media which can be addressed by adopting the self-sensing platform.

The amount of absorbed mass can be quantitatively calculated implementing the mathematical modeling framework presented in Sections 2 and 3. Comparison of the experimental results to those obtained from Sections 2 and 3 verifies the accuracy of the mathematical models.

**Figure 12.**
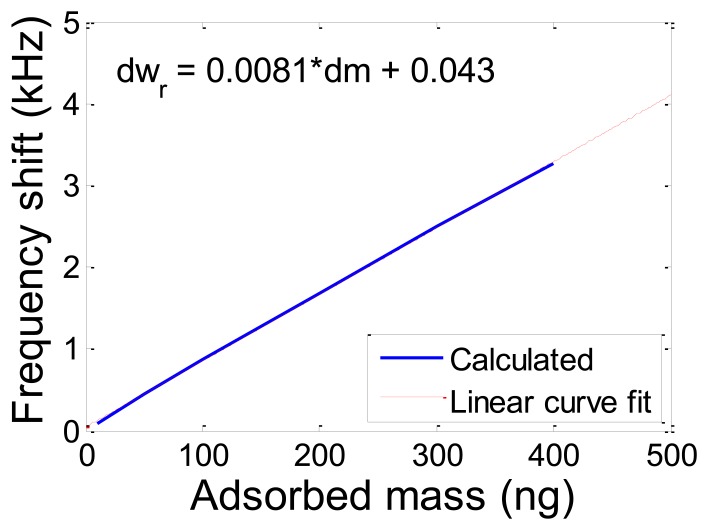
Quantification of frequency shift as a result of adsorbed mass exploiting mathematical modeling framework.

Adopting the mathematical modeling presented in this study the amount of adsorbed masses can be quantified having the shifts in resonance frequency. Figure 12 shows the shift in the first resonance frequency as a result of adsorbed mass utilizing the mathematical modeling framework providing the relationship between the added mass and frequency change.

The amount of adsorbed mass measured with self-sensing circuit and laser vibrometer was calculated to be 486.04 ng and 450.24 ng, respectively, utilizing this method of quantification. The sensitivity of the reported platform was measured to be about 122 pg/Hz.

## 5. Conclusions

A unique laser-less MC-based sensor which utilizes a Veeco Active Probe as a piezoelectric MC with self-sensing capabilities was proposed in this study. A pure capacitive bridge was designed to implement the detection platform in the self-sensing mode where the system was excited by applying a harmonic voltage to the piezoelectric layer which simultaneously produces output voltage as a result of the system's response. Utilizing the proposed platform, one self-sensing bridge can be exploited for both exciting the system and measuring the response of the system, thus eliminating the need for bulky and expensive optical based detection techniques.

Three main sections were presented for proving the concept of self-sensing methodology and testing its capability to be used as a mass sensing platform. A comprehensive distributed-parameters modeling framework was proposed for the self-sensing MC biosensor performing in dynamic mode. Since piezoelectric properties of material vary at the nanoscale, an adaptation law was exploited in order to compensate for the changes of piezoelectric properties of the ZnO stack embedded in the active probe. Numerical simulations were carried out in Matlab and presented. It was shown that the level of accuracy for measuring the fundamental resonance frequency of MC increases from 97.48% to 99.82% using adaptation strategy. In order to utilize the platform for mass sensing purposes, the capability of measurement system was compared and verified with optical-based read-out and a 99.63% accuracy was illustrated.

Implementing the proposed platform as a biological sensor, an extensive experimental setup was built to detect thiol groups immobilized over the MC surface. The shift in the first resonance frequency as a result of mass adsorption was obtained through both optical and self-sensing methods indicating the immobilization of mass over the MC surface.

The present study paves the way towards implementing such a system for detection of the concentration of any type of biomolecules and further developing a laser-less, cost-effective and portable diagnostic kit for any biomarker protein or biomolecule. It is planned to improve the proposed platform with higher sensitivity and selectivity for detection of smaller proteins such as PSA and myocardial infarction marker proteins, and also hybridization of DNA with the implementation of sensor and reference MC in the diagnostic platform.

## Figures and Tables

**Table 1.** The system parameters used for modeling.

**Parameters**	**Value**	**Units**
*L*	486	μm
*L_1_*	325	μm
*L_2_*	360	μm
*b*	50	μm
*t_b_*	4	μm
*t_p_*	4	μm
*ρ_b_*	2,330	kg·m^−3^
*ρ_p_*	6,390	kg·m^−3^
*E_b_*	105	GPa
*E_p_*	104	GPa
*d_31_*	11	pC/N

**Table 2.** Comparing the results obtained from mathematical modeling presented in parts I and II with the experimental results.

	**First Natural Frequency,*w****_n1_***(kHz)**	**Precision (%)**
Theory Section 2: Self-sensing	52.9	97.48
Theory Section 3: Self-sensing, Adaptive estimation	51.6	99.82
Experiment: Self-sensing	51.50	99.63
Experiment: Laser vibrometer	51.69	—
